# Do Exact Calculation and Computation Estimation Reflect the Same Skills? Developmental and Individual Differences Perspectives

**DOI:** 10.3389/fpsyg.2018.01316

**Published:** 2018-07-27

**Authors:** Dana Ganor-Stern

**Affiliations:** Achva Academic College, Arugot, Israel

**Keywords:** numerical cognition, computation estimation, exact calculation, development, multi-digit arithmetic, individual differences

## Abstract

Groups of children in 4th, 5th, and 6th grades and college students performed exact calculation and computation estimation tasks with two-digit multiplication problems. In the former they calculated the exact answer for each problem, and in the latter they estimated whether the result of each problem was larger or smaller than a given reference number. The analyses of speed and accuracy both showed different developmental patterns of the two tasks. While the accuracy of exact calculation increased with age in childhood, the accuracy of the estimation task reached its maximum level already in 4th grade and did not change with age. The reaction time of the exact calculation task was longer than that of the estimation task. The reaction time for both tasks remained constant in childhood and decreased in adulthood, with the improvement in speed larger for the exact calculation task. Similarly, within group variability in accuracy was larger in the exact calculation task than in the computation estimation task. Finally, low correlation was found between the accuracy of the two tasks. Together, these findings suggest that exact calculation and computation estimation reflect at least in part different skills.

## Introduction

The present study focuses on the ability to solve multi-digit multiplication problems exactly and approximately. Children learn in school to solve arithmetic problems exactly. It has been shown that in the early stages of multiplication skill acquisition children use various calculation techniques to solve single digit (1D) multiplication problems (e.g., Siegler, 1988; Koshmider and Ashcraft, 1991). Similar to the process that occurs for single digit addition problems, with practice children gradually shift to solving such problems through retrieval from memory (e.g., Ashcraft and Battaglia, 1978; Ashcraft, 1992; LeFevre et al., 1996). Such a strategy shift is assumed to be due to an associative network stored in long term memory that includes the single digit multiplication or addition problems together with their respective answers (e.g., Siegler, 1988; Koshmider and Ashcraft, 1991). The formation of this associative network depends on an extensive practice with such problems and thus it is more likely to be formed when the problem set is small, as in the case of single digit multiplication or addition problems (e.g., Zbrodoff and Logan, 1986; Logan and Klapp, 1991).

Much less research was devoted to the investigation of how multidigit multiplication problems are solved (e.g., van der Ven et al., 2015). The number of multidigit numbers is substantially larger than the number of single digit numbers, and therefore the number of multiplication problems composed of such numbers is also greater. Each problem will thus receive less practice, and this will lead to weak, if any, associations between the problems and their respective answers (Siegler, 1988; Koshmider and Ashcraft, 1991). Thus, although the ability to solve complex multi-digit arithmetic problems via calculation improves with age during childhood and from childhood to adulthood, as indicated by increased accuracy and speed (Jordan et al., 2003; Ulf, 2010), such problems in large will not be solved in any stage via retrieval, but rather through the application of multi-step calculation algorithms, that rely heavily on working memory (Lee and Kang, 2002; DeStefano and LeFevre, 2004; Kalaman and Lefevre, 2007; Raghubar et al., 2010). Due to working memory limitations people often use paper and pencil, or even turn to calculators.

In many real life circumstances it is sufficient to produce approximate, rather than exact answers to complex arithmetic problems such as multi-digit multiplication problems. For example, when planning a wedding party one might be interested in the approximate rather than exact cost involved in inviting 130 people, with the price of catering being 27$ per person. The process of producing an approximate answer to an arithmetic problem is called computation estimation (Rubinstein, 1985). Its main advantage is that it takes less time and attentional resources than exact calculation, and thus can be used in circumstances where time or attention resources are limited. It should be noted that the importance of computation estimation is not undermined by the wide use of calculators, as using a calculator to solve a multidigit problem is prone to typing errors, and computation estimation can be used as a sanity check to quickly evaluate whether the calculator generated answer is reasonable (LeFevre et al., 1993; Siegler and Booth, 2005).

Despite its importance, computation estimation has received relatively little attention in the educational system and in the numerical cognition literature (e.g., Siegler and Booth, 2005). One way to investigate this skill is using the estimation production task, in which participants are asked to produce an approximated answer for an arithmetic problem (e.g., LeFevre et al., 1993; Dowker et al., 1996; Lemaire and Lecacheur, 2002; Imbo and LeFevre, 2011). It has been shown that the accuracy of such approximated answer improves with age, although it is still poor even for adults (e.g., LeFevre et al., 1993). An examination of the strategies used based on participants self reports, reveals the use of various rounding techniques (e.g., Lemaire and Lecacheur, 2002; Lemaire et al., 2004; Siegler and Booth, 2005). With age there is an increased use of the more complex rounding strategies, and more adaptivity in strategy selection, such that the rounding procedure that introduced the least amount of error is chosen more often (e.g., Lemaire and Lecacheur, 2011). With age there is also more frequent use of post-compensation procedures to correct for the error introduced by the rounding procedures (e.g., Siegler and Booth, 2005).

In the estimation comparison task, another experimental paradigm used to study computation estimation in the context of multi-digit arithmetic, a multidigit multiplication problem was presented together with a reference number, and participants were required to estimate whether the exact answer to the problem was larger or smaller than the reference number (Ganor-Stern, 2015, 2016, 2017; Ganor-Stern and Weiss, 2015). The reference number was either far or close to the exact answer. The advantage of this task is that it enables the use of two distinct strategies. The first is the *approximated calculation strategy*, which involves rounding procedures, is the strategy mainly used in the estimation production task. The second is the *sense of magnitude strategy*, which involves an intuitive sense of magnitude without any calculation and it can be used only in this task due to the presence of the reference number. This strategy probably reflects the life-long experience with solving arithmetic problems and even the practice provided by the experimental session (Ganor-Stern and Weiss, 2015; Ganor-Stern, 2016). Past research has repeatedly shown adaptivity in strategy choice, as the approximated calculation strategy was used more often when the reference number was close to the exact answer, and thus the sense of magnitude cannot guarantee a correct response, while the sense of magnitude was used more often when the reference number was far from it. In terms of speed and accuracy it has been consistently shown that performance in this task is enhanced for reference numbers that are far vs. close to the exact answer, and for those that are smaller vs. larger than the exact answer (Ganor-Stern, 2015, 2016, 2017; Ganor-Stern and Weiss, 2015).

Ganor-Stern (2016) has investigated the developmental pattern of performance in this task looking at 4th graders, 6th graders and college students. There was some improvement in accuracy with age, as percent error was 22% for 4th graders and 17% for 6th graders and 16% for adults. This improvement was limited to trials in which the reference numbers were close to the exact answer; there was no improvement in accuracy for trials where the reference numbers were far from the exact answer. There was a substantial improvement in speed with age, especially in adulthood. Thus, while 4th and 6th graders took on average 12 and 11 s to respond, respectively, adults responded in only 4 s. In terms of strategy use, with age there was a decrease in the use of the sense of magnitude strategy and an increase in the use of the approximated calculation strategy, which presumably underlies the improvement in the accuracy for the close reference trials.

### Present Study

Despite the fact that estimation of the results of arithmetic problems is a useful skill in life it is still debated whether it reflects the same skill as solving the same problems exactly. This is the main question addressed by the current research. Research conducted on young children (between ages 5 and 9 years old) has shown positive relationship between the exact calculation and the estimation skills using addition problems (e.g., Dowker, 1997). Although when looking at children who show especially weak exact calculation skills, their estimates were found to be similar to those with average calculation skills, which implies some dissociation between the two skills (Dowker, 2005). In a similar manner, a study by Liu (2013) conducted on adults has shown that the problem size affected exact calculation but not estimation. Specifically, the larger the problem the higher the error rate and reaction time when participants solved it exactly, but not when they estimated its answer.

The present study expands past research by using a different estimation task than Dowker (1997) and Liu (2013), by looking at the developmental patterns of the estimation and exact calculation tasks from childhood to adulthood, at individual differences within each task and age group, and at the correlation between performance in the estimation and exact calculation tasks.

Specifically, groups of 4th graders, 5th graders, 6th graders, and college students solved a set of 20 2D multiplication problems exactly, and estimated the results of another set of 40 similar 2D multiplication problems relative to a reference number using the estimation comparison task (Ganor-Stern, 2015, 2016, 2017). In both tasks, speed and accuracy were analyzed by age. For the estimation task the analysis looked also at the effects of the reference number characteristics (its magnitude relative to the exact answer and its distance from the exact answer) on performance.

As to the predictions, on the one hand, one might expect a strong relationship between performance in the two tasks, as they both require arithmetic processing of the same stimuli (e.g., Dowker, 1997). On the other hand, past research provided evidence for dissociations between exact calculation and approximation, as exact calculation is language-dependent while approximation is not (e.g., Pica et al., 2004). Moreover, they seem to activate different areas in the brain. During exact calculation there is strong activation in the left inferior prefrontal cortex, while during approximation there is activation in the inferior parietal lobule in both hemispheres (e.g., Dehaene et al., 1999).

Furthermore, while the exact calculation task used in the current study involves a long working-memory-dependent algorithmic process, the computation estimation task seems to reflect a basic sense of magnitude together with a shortened algorithmic process (Ganor-Stern and Weiss, 2015; Ganor-Stern, 2016). Indeed, the results of a recent study on the effect of attention deficit hyperactivity disorder (ADHD) on estimation vs. exact calculation support some dissociation between exact calculation and the two strategies involved in the estimation comparison task. Participants with ADHD, which is assumed to involve working memory and executive function deficiencies (Castellanos et al., 2006), were impaired when conducting exact calculation and when using the approximated calculation strategy in the estimation task, but not when the sense of magnitude strategy was used (Ganor-Stern and Steinhorn, 2018).

As to development with age, based on past research that showed little improvement in estimation accuracy from childhood to adulthood (Ganor-Stern, 2016), but a significant improvement in the accuracy of exact calculation (e.g., Ulf, 2010) we expect to see more improvement with age in the accuracy of exact compared to approximated calculation. Speed is expected to increase in both tasks, although to a greater extent in the exact calculation task (e.g., Ulf, 2010). Finally, we expect to find more variability in performance (in accuracy or speed) across participants within each age group in the exact calculation compared to the estimation task (Dowker, 2005).

## Materials and Methods

### Participants

There were four groups of participants. Thirty three children from fourth grade (20 females), 33 children from 5th grade (16 females), 33 children from 6th grade (18 females), and 25 college students (23 females). The children were from three public schools in the center of Israel, and the college students were from a public academic college. The average age of the 4th graders was 9.8 years old, of the 5th graders it was 10.9 years old, of the 6th graders it was 12.04 years old, and of the college students it was 23.1 years old.

### Ethics Statement

The procedure was approved by the ethics committees of the Israeli Ministry of Education and of Achva Academic College, Israel. The college students provided written informed consent to participate in this study. Adhering to the policy of the Ministry of Education IRB, the parents of the school children denied consent by returning an enclosed form.

### Stimuli

The stimuli were 60 2-digit (D) multiplication problems. The problems in the estimation and in the exact calculation tasks were different, however, they were constructed with the same following restrictions. There were no tie problems. No operand had 0 as units digit. No reversed orders of operands were used (43 × 76 was not used with 76 × 43). The larger operand was on the left in half of the problems, and on the right in the other half. The problems for the estimation task were taken from Ganor-Stern (2016). The range of exact answers in the exact calculation task was 903–6391, and in the estimation task it was 768–8178.

The multiplication problems in both tasks were printed on sheets of paper. The exact calculation task included two sets of 10 problems each. Four problems were printed vertically on each page to leave space for the calculation. The estimation task that included 40 items was printed on a booklet. Each item was composed of a 2D multiplication problem with a reference number below it, and the word “smaller” written beneath the reference number on the left side, and the word “larger” written on the right side (Ganor-Stern, 2016). Four problems were printed on a sheet of paper. The reference numbers were of 4 types: (1) one which was about one fifth of the exact answer, (2) one which was about five times the exact answer, (3) one which was about one half of the exact answer, and (4) one which was about twice the exact answer. Ten problems were associated with each reference number type. Types (1) and (2) are the far condition, and types (3) and (4) are the close condition. In (1) and (3) the exact answer is larger than the reference number, and in (2) and (4) the exact answer is smaller than the reference number. Reference numbers were rounded to the nearest hundred. In half of the trials the exact answer was larger than the reference number, and in the other half it was smaller than the reference number.

### Procedure

The experiment took place in a class setting. The experimenter explained the participants that they will be solving 2D multiplication problems. The participants were first given a set of 10 2D multiplication problems printed on sheets of paper, and were instructed to solve the problems exactly on the paper sheets. Then they were given a booklet with 40 estimation items. Each item was consisted of a 2D multiplication problem with a reference number below it, and the word “smaller” on the left side, and the word “larger” on the right side. The participants were asked to indicate for each problem whether they estimated the exact answer to be smaller or larger than the given reference number by marking either the word “larger” or the word “smaller.” Finally, the participants were given a new set of 10 2D multiplication problems printed on sheets of paper to solve them exactly on the paper. There were no time limits. For each task, the experimenters documented on each participant’s sheet of paper the time he/she started each set of problems. The students were asked to raise their hands when they finished the current set. The experimenter documented the time the participant ended the task on the paper sheet, and handed him/her the following set. Participants were not allowed to use calculators in any of the tasks.

## Results

The performance measures for each task were the accuracy for each problem and the solution time for each problem set, which was divided by the number of problems, for an average solution time for a single problem. The analyses examined the developmental patterns within each task, the between-participants variability within each task, and the relationship between performance in the two tasks.

### The Developmental Pattern in the Exact Calculation Task

**FIGURE 1:**
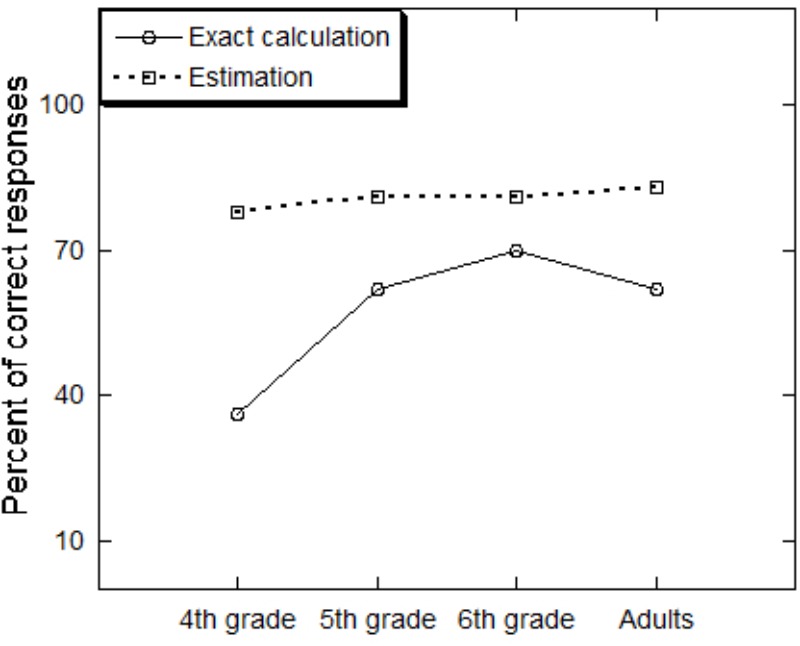
Percent of correct responses for the exact calculation and estimation tasks by age group.

**FIGURE 2:**
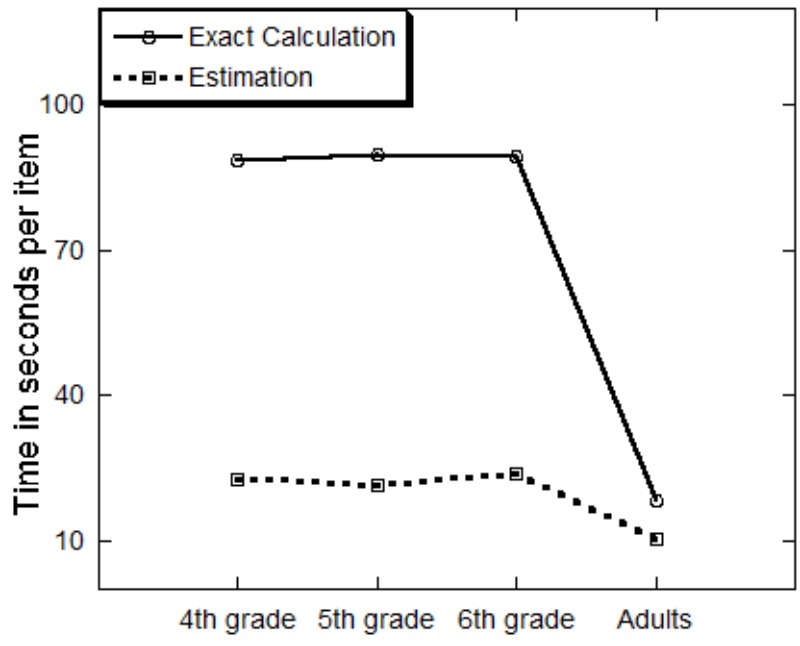
Average response time (in seconds) for the exact calculation and estimation tasks by age group.

A one way Analysis of Variance (ANOVA) on the percentage of correct responses in the exact calculation task with age as a between-participants variable has shown that percent of correct responses increased with age (*F*_3,118_ = 8.07, *MSE* = 8.49, *p* = 0.0001, ${\text{η}}_{\text{p}}^{2}$ = 0.17). Sheffe *post hoc* tests showed that 4th graders were less accurate (36%) than the other groups (*p* < 0.05), that did not differ (**Figure 1**). Percent of correct responses was 62, 69, and 62 for 5th graders, 6th graders and adults, respectively. The speed analysis revealed a significant effect of age (*F*_3,120_ = 24.40, *MSE* = 1386.7, *p* = 0.0001, ${\text{η}}_{\text{p}}^{2}$ = 0.38). Sheffe *post hoc* tests showed that the adults were faster than the children groups (*p* < 0.05), that did not differ. Average response time was 88.82, 90.00, and 89.46 s for the 4th graders, 5th graders, and 6th graders, respectively, and it was 18.12 s for adults (**Figure 2**).

### The Developmental Pattern in the Estimation Task

An ANOVA on the average response time has shown a significant effect of age (*F*_3,119_ = 9.72, *MSE* = 48.52, *p* = 0.0001, ${\text{η}}_{\text{p}}^{2}$ = 0.20). Again sheffe *post hoc* tests have shown that adults were faster than the children groups when solving the estimation task (*p* < 0.01), while the children groups did not differ in speed (**Figure 2**). Thus, while it took adults on average 10.2 s to respond to a problem, it took 4th graders about 22.73 s, 5th graders 21.5 s, and 6th graders 23.77 s.

The accuracy analysis included in addition to the age factor also the within-participant factors of the size of the reference number (larger vs. smaller than the exact answer) and its distance from the exact answer (far vs. close).^1^ As was found in past research with the same task (Ganor-Stern, 2015, 2016, 2017; Ganor-Stern and Weiss, 2015), accuracy was higher when the reference number was far (83%) compared to close (78%) to the exact answer (*F*_1_,_120_ = 19.66, *MSE* = 0.36, *p* = 0.0001, ${\text{η}}_{\text{p}}^{2}$ = 0.14). It was also higher when the reference number was smaller (83%) than the exact answer compared to when it was larger (79%) than it, although the effect was marginally significant (*F*_1_,_120_ = 3.55, *MSE* = 0.06, *p* = 0.06, ${\text{η}}_{\text{p}}^{2}$ = 0.03). Importantly, as can be seen in **Figure 1**, accuracy did not differ across the age groups (*F* < 1).

### Cross-Participants Variability in Performance in the Exact Calculation and Estimation Tasks

To examine cross-participants variability in performance the coefficient of variability was calculated for each age group and for each task, for accuracy and speed separately. This was done by dividing the standard deviation of accuracy and of speed across participants by the group average and multiplying by 100. The results (**Table 1**) show that the coefficient of variability in accuracy was higher for the exact calculation task compared to the computation estimation task, and that it decreased with age for the former but not for the latter. The coefficient of variability in speed does not show a consistent pattern across tasks or across age.

**Table 1:** Coefficient of variability in accuracy and speed by task and age group.

	Accuracy		Speed	

Age group	Exact calculation	Computation estimation	Exact calculation	Computation estimation
4th grade	77.72	15.64	47.73	44.99
5th grade	49.61	17.71	45.13	66.87
6th grade	35.01	20.00	44.14	40.69
Adults	51.44	17.79	38.32	36.02

### Relationship Between the Performance in the Exact Calculation and Estimation Tasks

To examine the relationship between the two tasks, we calculated the correlation between the accuracy of the two tasks and the reaction time of the two tasks. This was done separately for each age group, and across age groups (**Table 2**). The correlation between the accuracy of the two tasks, collapsed across the age groups, was 0.35 (*p* < 0.05), and between the speed of the two tasks was 0.60 (*p* < 0.05). As can be seen in **Table 2**, the pattern of stronger inter-task correlation in speed than in accuracy was found in most age groups. This is possibly due to the low variability in accuracy found in the estimation comparison task. Accuracy level in the estimation task showed the least variability across the age groups (**Figure 1**) and across- participants within each age group (**Table 1**).

**Table 2:** Inter-task correlation and reliability coefficients by task and age group.

	Inter-task correlation		Split-half reliability		

	Accuracy	Speed	Exact Calculation Accuracy	Exact Calculation Speed	Estimation Accuracy
All	0.35	0.60	0.87	0.88	0.79
4th grade	*0.35*	0.38	0.77	0.86	0.58
5th grade	0.36	0.62	0.88	0.78	0.89
6th grade	*0.13*	0.35	0.83	0.95	0.79
Adults	0.53	*-0.07*	0.89	0.92	0.84

*The numbers in plain font represent significant correlations (p < 0.05), while the numbers in italics and in a smaller font represent insignificant correlations.*

To examine whether the low inter-task correlation (at least in accuracy) is due to low reliability of the tasks, we calculated split half reliabilities for each of the tasks. As can be seen in **Table 2** the split half reliabilities of the two tasks were relatively high (in most cases they were higher than 0.80), thus suggesting that the inter-task correlations were not restricted by the tasks reliabilities^1^.

## Discussion

From a developmental perspective, accuracy in the exact calculation task improved from 4th to 5th grade and then remained unchanged up to adulthood. Note that percent of correct responses hardly reached 70%, far from perfect accuracy, thus suggesting that participants even in adulthood are not proficient in solving multi-digit multiplication problems, probably due to the wide use of calculators. Note that the accuracy level of the two tasks is not comparable as the exact calculation task is an open ended task, while the computation estimation task is a forced choice one. Thus, what seems to be informative is the different patterns across age. While exact calculation accuracy increased with age, accuracy of the computation estimation task did not change by age at all. As to speed, speed improved in both tasks mainly in adulthood, although the increase was much more pronounced for the exact calculation task.

Past research has found a continuous improvement in accuracy (van der Ven et al., 2015) and in speed (Koshmider and Ashcraft, 1991; De Brauwer and Fias, 2009) throughout childhood when solving single digit multiplication problems exactly. In the present study the only improvement in accuracy of exact calculation was found between 4th and 5th grades. The reason might lie in the difference between single vs. multiple digit multiplication. Single digit multiplication is practiced on its own, and as part of multidigit multiplication, and thus it continues to improve. Multidigit multiplication is practiced much less, in part due to the increased use of calculators.

The main improvement in speed is seen in adulthood. This is true for both tasks but it is more apparent for the exact calculation task. This improvement could be due to improvement of domain-specific skills, such as calculation skills (e.g., Pauli et al., 1994), or domain general factors, such working memory and decision processes (e.g., Berg, 2008). The fact that the improvement in speed in adulthood was not accompanied with an improvement in accuracy might suggest that domain general factors accounted for it.

Note that the accuracy levels in the estimation task of the current study are comparable to those found in past research using the same task and the same age groups (Ganor-Stern, 2016). The reaction time here are longer due to the use of paper and pencil, however, the speed patterns are similar. In both studies speed remained unchanged in childhood and it improved considerably in adulthood. The facts that similar patterns were found in the two studies both in accuracy and in speed despite the use of different procedures [i.e., the current study used a paper and pencil procedure, while in Ganor-Stern (2016) the experiment was computerized] provide convergent validity to the current results.

The different developmental trajectories of the two tasks suggest that they do not reflect the same skill. In a consistent manner, the analysis of variability has shown that the variability in accuracy was smaller for the computation estimation task than for the exact calculation task. Moreover, while for the exact calculation task this variability decreased with age, consistent with past research (De Brauwer and Fias, 2009), for the computation estimation task it did not. The relatively low correlation between the accuracy of the two tasks also corroborates the dissociation between the two tasks.

The present research showing an increase in accuracy between 4th and 6th grades in the exact calculation task is in line with Ulf (2010), who found a similar pattern. Note, however, that in contrast to the present findings, Ulf also reported an improvement in approximated calculation between 4th and 6th grades. A possible explanation for this difference is the nature of the estimation task used. In Ulf (2010) children were given addition and subtraction problems composed of 2D numbers presented vertically. Each problem was accompanied with two proposed answers, and the task was to choose the answer that was closest to the correct answer. Such a task might encourage participants to solve the problem exactly, and thus might show similar improvements with age for the exact calculation and estimation tasks.

The current estimation comparison task seems to capture not only approximated calculation but also sense of magnitude for the results possible for such multidigit multiplication problems. This is indicated by the use of sense of magnitude strategy itself, and by the adaptive choice between the sense of magnitude and the approximated calculation strategies. This sense of magnitude might be related to the Approximate Number System (ANS), which represents magnitudes in an approximated manner, develops early, and is language independent (Ansari, 2008; Mazzocco et al., 2011a,b; Park and Brannon, 2013).

Across studies and age groups participants use the approximated calculation strategy more often when the reference numbers are close to the exact answer than when they are from it, suggesting that participants have a rough sense for how big the answers could be, and thus use the approximated calculation strategy more often when the reference number is within this range and the sense of magnitude strategy when the reference number it is outside of it (Ganor-Stern, 2015, 2016, 2017; Ganor-Stern and Weiss, 2015). Importantly, this pattern of adaptive strategy choice was found even for children as young as 4th graders (Ganor-Stern, 2016) and for adults diagnosed with developmental dyscalculia (Ganor-Stern, 2017).

The conclusion of the current study that exact calculation and estimation do not reflect the same skill is consistent with past research that argue for a dissociation between estimation and exact calculation (e.g., Pica et al., 2004; Liu, 2013), more generally it is compatible with theories that emphasize the componential nature of arithmetic (e.g., Dowker, 2005).

The current study did not collect information about strategy use. Future research, in which participants describe the strategy they used on a trial by trial basis should look at the relationship between exact calculation and estimation performance separately for the two strategies used. It is predicted that the correlation between the accuracy of the estimation and exact calculation tasks will be higher for trials in which the approximated calculation strategy was used.

### Limitations of the Present Study

The fact that the computation estimation task was a forced choice task, and the exact calculation task was an open ended one prevents a direct comparison of the accuracy and speed of the two tasks, and this might be seen as a limitation of the current study. The rational for this design is that the use a forced choice task with reference numbers in the estimation task allowed using sense of magnitude when solving this task, especially with far reference numbers. For the exact calculation task, the use of a forced choice task might have encouraged participants to use shortcut strategies, such as parity rules, (e.g., Lemaire and Fayol, 1995) rather than to go through the whole solution process, and thus an open ended format was used. Furthermore, the task order was determined by school considerations, which did not allow for a random or counterbalanced design. As a consequence no conclusions on the effect of performing one task on the other task can be drawn. Finally, the measurement of speed was possible for the whole set rather than for each item, due to the use of paper and pencil, rather than a computerized task. This was done because solving complex multidigit multiplication problems is usually done in everyday life with paper and pencil, and the experimental setting tried to mimic these natural conditions.

## Author Contributions

DG-S conception and design, statistical analyses, interpretation of data, and drafting the manuscript.

## Conflict of Interest Statement

The author declares that the research was conducted in the absence of any commercial or financial relationships that could be construed as a potential conflict of interest.

## Footnotes

1 As the estimation task included only one problem set, we had an accuracy measure for each item and a speed measure for the whole set. This enabled us to analyze the effect of reference number characteristics and to calculate the split half reliability of the estimation task for accuracy only, and not for speed.
